# Autopolyploidy genome duplication preserves other ancient genome duplications in Atlantic salmon (*Salmo salar*)

**DOI:** 10.1371/journal.pone.0173053

**Published:** 2017-02-27

**Authors:** Kris A. Christensen, William S. Davidson

**Affiliations:** Molecular Biology and Biochemistry, Simon Fraser University, Burnaby, British Columbia, Canada; Universite de Rouen, FRANCE

## Abstract

Salmonids (e.g. Atlantic salmon, Pacific salmon, and trouts) have a long legacy of genome duplication. In addition to three ancient genome duplications that all teleosts are thought to share, salmonids have had one additional genome duplication. We explored a methodology for untangling these duplications from each other to better understand them in Atlantic salmon. In this methodology, homeologous regions (paralogous/duplicated genomic regions originating from a whole genome duplication) from the most recent genome duplication were assumed to have duplicated genes at greater density and have greater sequence similarity. This assumption was used to differentiate duplicated gene pairs in Atlantic salmon that are either from the most recent genome duplication or from earlier duplications. From a comparison with multiple vertebrate species, it is clear that Atlantic salmon have retained more duplicated genes from ancient genome duplications than other vertebrates--often at higher density in the genome and containing fewer synonymous mutations. It may be that polysomic inheritance is the mechanism responsible for maintaining ancient gene duplicates in salmonids. Polysomic inheritance (when multiple chromosomes pair during meiosis) is thought to be relatively common in salmonids compared to other vertebrate species. These findings illuminate how genome duplications may not only increase the number of duplicated genes, but may also be involved in the maintenance of them from previous genome duplications as well.

## Introduction

Atlantic salmon belong to the family Salmonidae, which also includes the Pacific salmon (e.g. Chinook salmon–*Oncorhynchus tshawytscha*), chars (e.g. Arctic char–*Salvelinus alpinus*), trout (e.g. rainbow trout–*Oncorhynchus mykiss*), graylings, and whitefishes. The ancestral species of the salmonids experienced a whole genome duplication not shared by other modern fishes [1]. This genome duplication (commonly referred to as the 4R genome duplication) is believed to have been a within species event (i.e. autopolyploidy).

The autotetraploid genome duplication in the Salmonidae family is thought to have occurred around 88 million years ago [1–4]. During the last 88 million years, it appears that many of the homeologous genes (paralogous/duplicated genes originating from a whole genome duplication also known as ohnologs) have been retained. In rainbow trout, around 25% of these copies were still found in the transcriptome [5] and 52% remained in the genome [3]. In the Atlantic salmon the percent of genes that remained in duplicate after the salmonid specific genome duplication was 55% [4].

Gene expression ratios may partially explain why some duplicated genes are retained. The loss of one of the genes would alter the expression ratio of that gene compared to the rest of the genome, which may impair fitness (other mechanisms may also explain gene duplicate retention, reviewed in [6]). In polyploid organisms, homeologous chromosomes pairing at meiosis may also maintain duplicated genes by homogenizing regions of the chromosomes that exchange DNA (reviewed in [7]). Polysomic inheritance has been extensively characterized in salmonids (reviewed in [8]).

Another artifact of autotetraploidy, besides generating similarity between homeologous genomes, may be the maintenance of existing homeologous gene pairs from more ancient genome duplications. Around 300 million years ago, another genome duplication, often referred to as the teleost specific genome duplication or the 3R genome duplication, occurred in the ancestor of nearly every fish species [9,10] (reviewed in [11]). Signs of this more ancient genome duplication may be better preserved in autotetraploids due again to polysomic inheritance. If a gene copy from the teleost-specific genome duplication was lost, polysomic inheritance in an autopolyploid species might replace that lost gene with a copy from a homeologous chromosome [12].

Alternatively, since there were potentially multiple copies of a gene from the teleost specific genome duplication, the multiple copies would then be doubled again during the salmonid specific genome duplication. With up to four copies of a gene, it may be that the more ancient gene duplicates are maintained simply by chance (i.e. a gene duplication is more likely to be retained from the teleost specific genome duplication after the salmonid specific genome duplication because there are now four copies of the original).

A major finding from the sequencing of the Atlantic salmon genome was that 20% of the genes duplicated in the teleost specific genome duplication were retained compared to 12–24% in other species [4,13]. This estimate suggests that the retention of teleost specific gene copy was similar between salmonids and other teleost species. It also suggests that polysomic inheritance may not conserve ancient homeologous gene pairs.

An additional two genome duplications presumably occurred before the divergence between the lamprey and the other vertebrates approximately 500 million years ago [10,14,15]. These duplications are shared by the salmonids, other fishes, and tetrapods (e.g. humans). These early duplications are much older and difficult to clearly identify [16] because the sequences of paralogs have diverged so much and many duplicated gene were lost.

With four genome duplications, understanding one duplication requires that it can be distinguished from the others. In order to investigate if autopolyploidy maintains evidence of previous genome duplications, we first needed to be able to distinguish one genome duplication from the rest. We present a novel methodology for doing so, and present evidence that autopolyploidy in Atlantic salmon maintains signatures of earlier genome duplications.

## Materials and methods

To identify duplicated genes, a new annotation of the Atlantic salmon genome was used. The method presented below is less stringent than most annotation pipelines. Many of the genes presented might be psuedogenes (likely very few), but the position was more important to our research question than whether a gene was functional or not. This methodology also allowed a standardized nomenclature for genes that was useful in comparisons between species and between chromosomes.

### Homeologous region detection

The following procedure was used to find homeologous regions in all organisms used in this study. A reference protein dataset was created by downloading all the sequences from the NCBI's RefSeq database matching the criteria, “((zebrafish) AND "Danio rerio"[porgn:__txid7955])” on 17 July 2014. Partial sequences and redundant proteins were removed from this reference using a custom Perl script (all scripts can be found in the S1 Appendix. The zebrafish protein dataset was chosen because it is well annotated and comprehensive. All reference genomes were downloaded from Ensembl except the Atlantic salmon genome. The Atlantic salmon was available from the NCBI (NC_027300-NC_027328).

**Fig 1:**
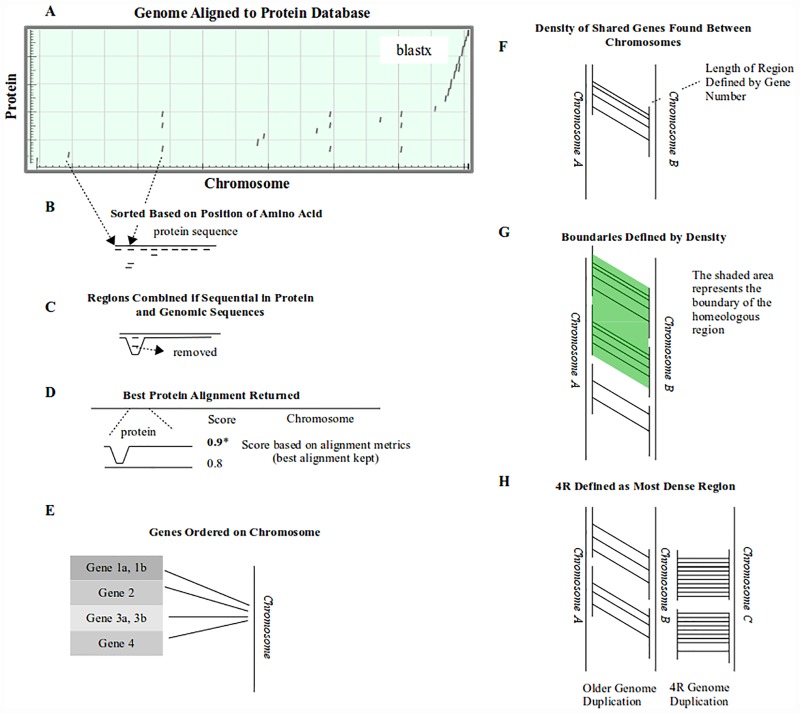
Representation of gene modeling and homeologous region identification. (A) The genomes of several organisms were aligned to the zebrafish protein database using BLASTX. (B) The partial alignments were sorted based on their alignment to the protein sequence. (C) Individual alignments of a protein were sorted and brought together if it was logical to do so (i.e. the alignments were in order relative to the genome and the protein sequences). Often, partial alignments would be found for multiple chromosomes. Many of these partial alignments were removed because there was not sufficient evidence to support a full alignment of the protein at that genomic location. (D) These gene models were then scored based on alignment scores and the percent of the protein that was represented in the gene model. If more than one gene model aligned to the same genomic location, the score was used to determine which was a better fit (or both were kept if they had a similar score). (E) The gene models were then sorted and the information was converted to gff3 format. (F, G) Homeologous regions were found using the density of homeologous gene models found between the different genomic regions. (H) For the Atlantic salmon, the 4R genome was differentiated from the rest of the genome duplications by identifying the most dense relationship in a region.

BLASTX [17,18] was used to align genomic sequences to the reference protein dataset with an evalue cutoff of 1e-4 and with the low-complexity filter turned off. Introns can be quite large in vertebrates [19] and consequently alignments are expected to produce only local alignments to exons. Further, misassembly of the genome may increase the intronic regions between exons. This typically creates a problem in gene modeling [19]. We addressed this issue with the methodology shown in Fig 1.

Using Perl scripts, local alignments were sorted based on their amino acid position. Only the best alignment of overlapping chromosomal alignments was retained. Local alignments were combined if they were sequential both at the amino acid and nucleotide level, and if they satisfied the following conditions: they were within 50K nucleotides of each other, within 300 amino acids of each other, and they did not overlap by more than 40 amino acids.

To reduce the number of paralogous sequences from aligning to the same chromosomal locus, a score was given to each overlapping gene model and only the best was retained. The score was calculated by multiplying the percent of the length of a protein reference sequence represented in a gene model by the average sequence alignment score produced by BLASTX. If the best overlapping gene models were within 0.05 units of each other, they were retained. A threshold of 0.3 was used as a cutoff value for the gene models (to remove psuedogenes). Gene models were then converted to GFF3 format relative to the genome, essentially ordering them on the chromosomes.

Homeologous regions of the genome were identified by comparing each gene model in a pairwise fashion and then identifying the regions with shared gene models. The boundaries of these regions were defined by the density of shared gene models. Four parameters were used when defining the boundary, including: *window*-the number of gene models to scan at a time, *density*-the fraction of homeologous genes within a window, *minimum windows*-the number of windows needed for homeologous regions to be called, and the *boundary*-the number of windows with low density to define the edge of a homeologous region. Overlapping homeologous regions were separated in Atlantic salmon based on density and region size (the highest density region was assumed to be from the salmonid specific genome duplication).

The older genome duplications were similar to each other, in terms of sequence similarity and density of homeologous genes in the genome, and we were unable to partition them into discrete units (personal observation). This means that the analyses for the proportion of observed synonymous substitutions (Ps) and homeologous gene density below include all of an organism's genome duplications not just the most recent, except for the Atlantic salmon's salmonid specific genome duplication. The relationships between homeologous chromosomes were visualized using circos plots [20] and the Integrative Genomics Viewer [21,22]. Various parameters were used for different species based on a visual inspection of the circos plots (see Table 1). If the same criteria were used, homeologous regions would be missed in some species (personal observation).

**Table 1:** Parameters used when defining homeologous regions.

	Atlantic_3R	Atlantic_4R	Chicken	Gar	Human	Zebrafish
**Window**	10	10	10	15	10	15
**Homeologous Genes in Window**	1	3	1	1	1	1
**Threshold for Including Region (count of the number of windows)**	10	10	10	8	20	8
**Boundary (number of windows that did not meet criteria on either side of region)**	9	9	9	9	8	9

### Ps estimation and gene density between homeologous regions

Using Perl scripts, homeologous relationships between gene models were defined as described above and the proportion of observed synonymous substitutions (Ps) for each of the shared gene models was found. The raw score for synonymous substitutions was used because these sequences are so diverged that they are expected to have reached mutational saturation and corrections for reverse mutations, for current models (KaKs Calculator [23]), may increase the synonymous substitution rate above 1. This makes the interpretation of the value problematic, and if one assumes a similar reverse mutation rate in all the compared organisms, it is unnecessary to make this correction.

In order to find the Ps for each pairwise relationship between the gene models, the following procedure was used: 1) the overlapping sequences were extracted from the genome, 2) each sequence was then aligned (BLASTX -culling_limit 1) to the protein sequence used to create the gene model, 3) the protein sequences from each of these alignments were then aligned to each other, 4) if there was a single alignment result, each protein sequence was then aligned back to the genome, 5) if the amino acid number and the nucleotide number matched, equal length pairs were used to calculate a portion of the total Ps value for each gene model using the program SNAP (“HIV Databases”; [24]), 6) The average Ps (if a gene model had multiple exons) was calculated for the gene model pair.

The density of homeologous genes was found by taking the homeologous gene count and dividing the count by the distance of the homeologous region (and then multiplied my 1MB to create a standardized value). In order to compare the densities between organisms, it was necessary to find the overall gene density for each region because the gene density might influence the homeologous gene density. The homeologous gene density for each region was divided by the total gene density to create a fraction for each region. The average of these fractions was compared between organisms. Gene density and Ps values were compared between species using a two-tailed, Welch's t-test. Ribosomal protein genes were high in the human genome and so these analyses were repeated with and without ribosomal proteins (proteins with “rp” as the first letters in the protein symbol).

To investigate the relationship between the homeologous gene density and the Ps value for individual homeologous regions, homeologous regions were identified similar to the method described above, but without regard to partitioning the regions from different genome duplications. The parameters used in identifying regions was uniform for this analysis for all species (window = 10, density = 1, boundary = 10). For each of these regions, the Ps values and homeologous gene densities were found as above and plotted on a scatter-plot. Several genomes were added for this analysis (chimpanzee, dog, stickleback, and tetraodon).

## Results

**Fig 2:**
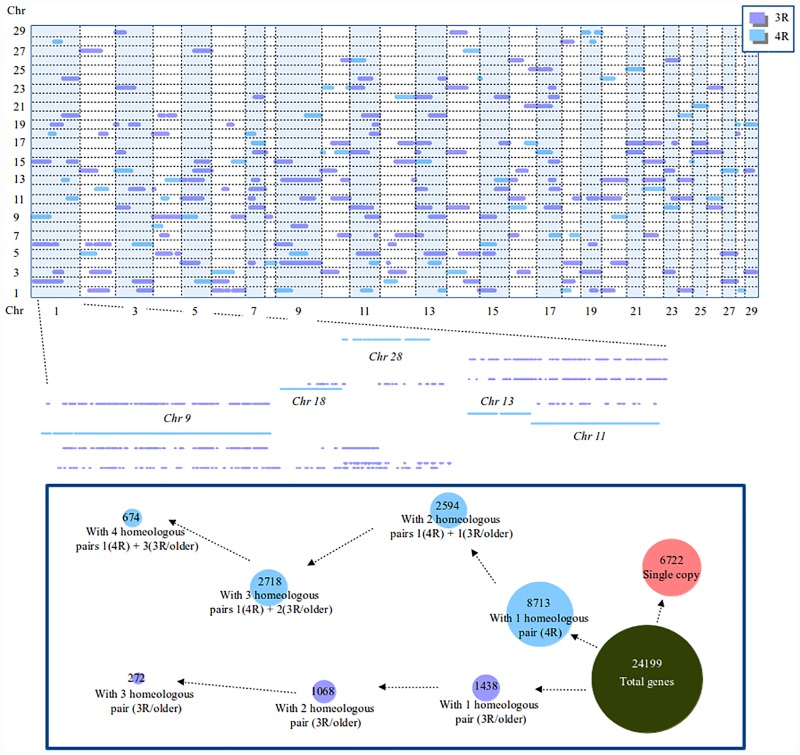
Plot of paralogous genes along specific Atlantic salmon chromosomes. The x-axis represents the Atlantic salmon genome, while the y-axis represents the corresponding homeologous gene pairs from homeologous regions of different Atlantic salmon chromosomes. The blue dots represent the 4R genome duplication and the purple dots represent older genome duplications. A larger representation of the first chromosome allows finer details to be seen. For example, it can be seen that for each 4R homeologous region, there appears to be three other older homeologous regions. The insert shows how the gene models are represented in the Atlantic salmon genome. There are a total of 24,199 gene models and 8,713 of them have a single corresponding 4R homeologous gene pair. An additional 2,594 gene models have a corresponding 4R homeologous gene pair and an additional 3R (or older) homeologous gene pair, 2,718 gene models have two additional 3R homeologous gene pairs, and 674 have three additional homeologous gene pairs. The remaining homeologous gene pairs do not have a 4R homeologous gene pair.

**Fig 3:**
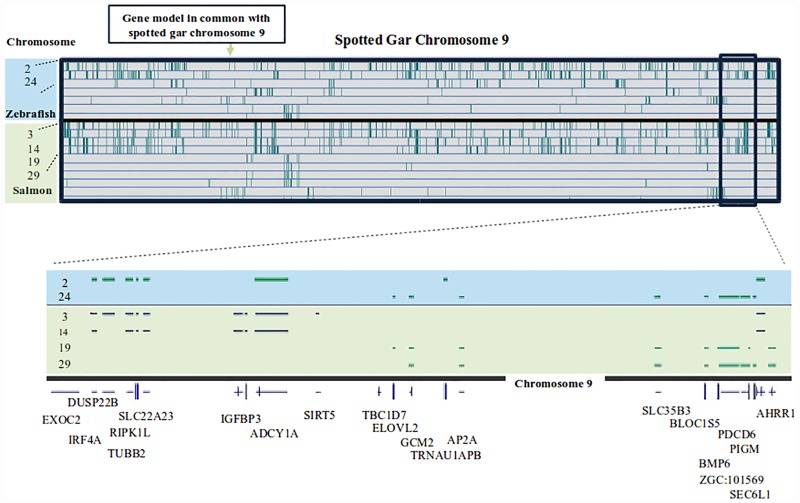
Small-scale example of homeologous regions (spotted gar chromosome 9). In this example, gene models were compared between organisms to identify the various genome duplications in different organisms. The spotted gar chromosome 9 was used to identify synteny between the gar, zebrafish, and Atlantic salmon. In the top portion, different chromosomes from zebrafish and Atlantic salmon are compared to chromosome 9 of the spotted gar. In this section, a vertical green line represents a matching gene model between the two organisms. Two of the zebrafish chromosomes share a large number of gene models (2 and 24). Four of the Atlantic salmon chromosomes show extensive synteny with the gar chromosome (3, 14, 19, and 29). In the lower section, a segment of the gar chromosome is highlighted with the genes in the region shown below. The zebrafish and Atlantic salmon syntenic genes are shown above the gene annotations. This example was selected because it illustrates the teleost specific genome duplication (in zebrafish, but not in the gar that split from the teleosts before the genome duplication), the salmonid specific genome duplication (in the Atlantic salmon), and how genes may be retained or lost after genome duplication.

**Fig 4:**
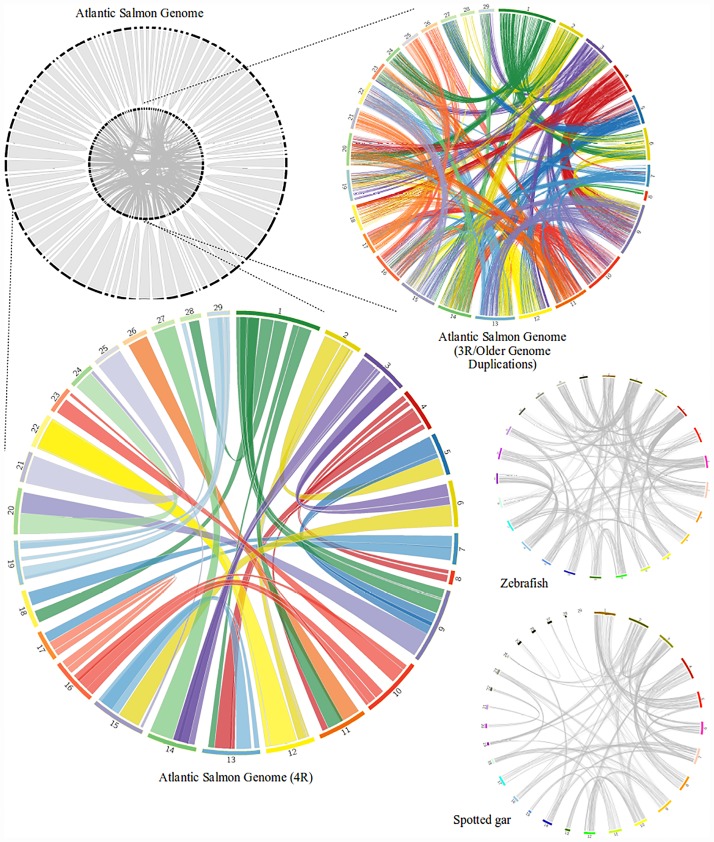
The Atlantic salmon genome duplications. The salmonid specific genome duplication is visualized along with older genome duplications for the Atlantic salmon in different circos plots. The ribbons, in the circos plots, represent the homeologous regions of the salmonid specific genome duplication and the lines represent individual homeologous gene-pairs of the teleost duplication and possibly older duplications. The zebrafish and gar genomes are included for comparison.

Based on the zebrafish protein dataset used in this study, a total of 58,362 gene models (referred to as genes below) were produced at 24,199 unique genomic locations in the Atlantic salmon genome (Fig 2). Of these, 15,729 were non-redundant gene models (~27%). A fine-scale example can be seen in Fig 3. Clear relationships between homeologous regions were found for the Salmonidae specific genome duplication (4R) and previous genome duplications (referred to as the 3R genome duplication below, for simplicity) (Figs 2 and 4). The Salmonidae specific genome duplication presented here is very similar to that found in Lien *et al*. (2016). The teleost specific genome duplication and previous genome duplications could not be compared because this has not previously been examined. The zebrafish and spotted gar genomes are also shown in Fig 4, and either the teleost specific or more ancient genome duplications can be seen. Often, a segment from a single zebrafish chromosome shared homeologous genes between several other chromosomes; perhaps evidence for both the teleost specific genome duplication and more ancient genome duplications--possibly from segmental duplications. The human and chicken circos plots are shown in S1 Fig. Both show extensive evidence for ancient genome duplications.

**Fig 5:**
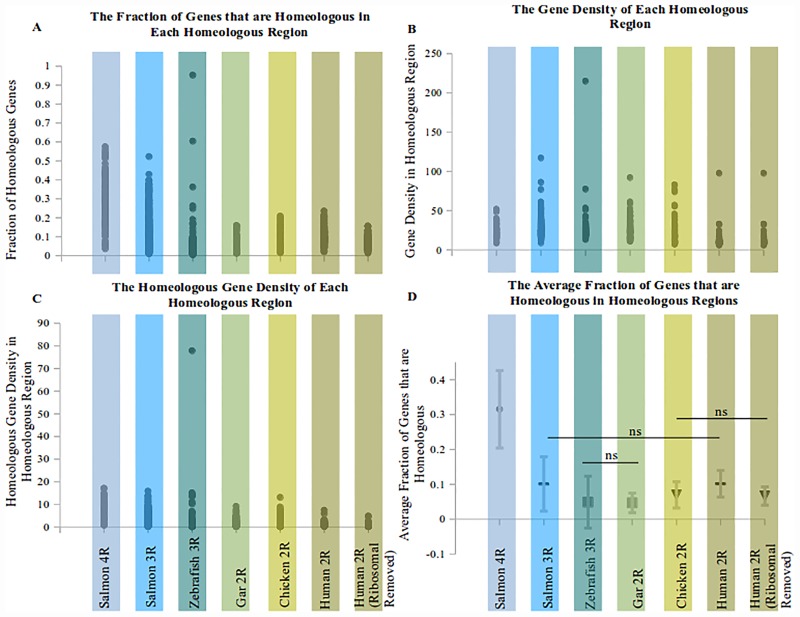
Homeologous gene density in various vertebrate species. (A) The plot displays the homeologous gene density for homeologous regions in the Atlantic salmon and other organisms (i.e. (Average homeologs per 1Mb)/(Average number of genes per 1Mb) = Average of the fraction of genes that are homeologous per region). (B) The gene density in identified homeologous regions per 1 million base pairs. (C) The homeologous gene density in the same identified homeologous regions per 1 million base pairs. (D) The average homeologous gene density corrected by dividing the homeologous gene density by the gene density (ns = not significant, all other comparisons are significantly different p < 0.05, bars are the standard deviation of the homeologous regions' corrected homeologous gene density).

An analysis of the homeologous gene density among several vertebrates is presented in Fig 5. The salmonid specific genome duplication (4R) has the greatest density of homeologous gene pairs of all the genomes (Fig 5D). Interestingly, the teleost specific (3R) duplication/older genome duplications in Atlantic salmon appears to be better conserved than the same teleost specific genome duplication in the zebrafish (p-value < 0.01, Fig 5D). Only the density of homeologous gene pairs in the human were as high as the Atlantic salmon's teleost specific genome duplication, but ribosomal proteins appeared to be the reason for this observation (Fig 5D and S1 Fig). If ribosomal proteins were removed from the analysis, the human homeologous gene pair density was similar to the chicken's.

Surprisingly, the teleost specific genome duplication (3R) in zebrafish appears to have very similar conservation of homoleogous gene pairs to organisms that only experienced the two genome duplications common to other vertebrates (Fig 5D). It should be noted that there is a segment of the zebrafish chromosome 4 that appears to have a high density of homeologous gene pairs (Fig 5A–5C). This region contains ribosomal proteins and has been observed before [25]. This did not greatly influence the comparison in Fig 5D because the gene density in this region is also quite high and so the fraction of homeologous genes in this region was not very high.

**Fig 6:**
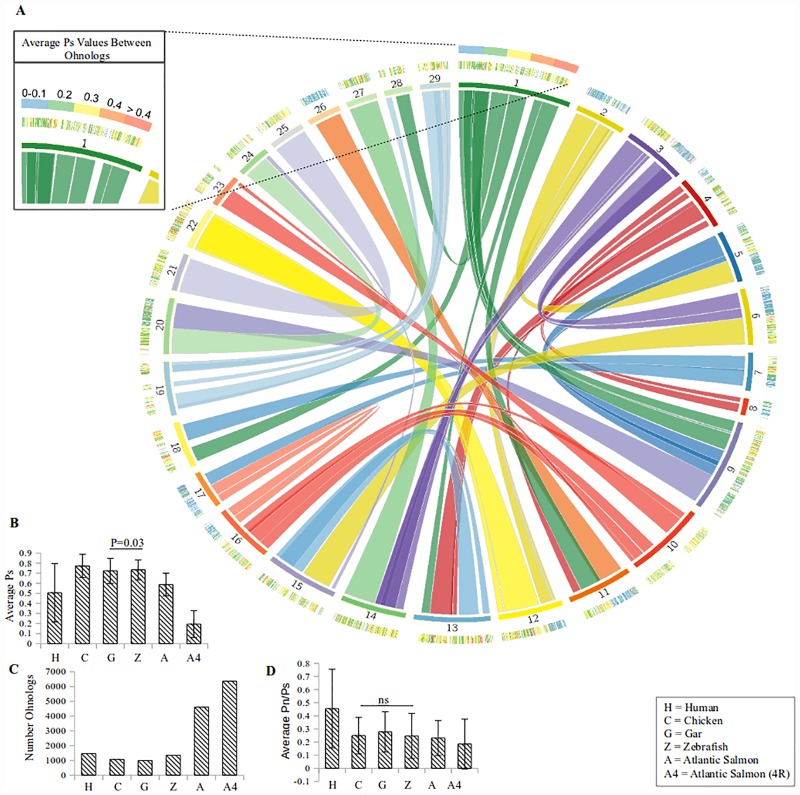
Proportion of silent substitutions (Ps) between homeologous gene pairs. (A) Circos plot of the homeologous regions from the Salmonid specific (4R) genome duplications with associated Ps values on the outer perimeter. (B) The average Ps value for all gene pairs, found in homeologous regions, of a species (all comparisons were significantly different p < 0.05). (C) The number of gene pairs in homeologous regions (includes reciprocal gene pairs). (D) The average Pn/Ps values for all homeologous gene pairs for the different species (only the chicken and zebrafish were not significantly different).

The proportion of observed synonymous substitutions (Ps), often a proxy for neutral mutation and time, was found between homeologous gene pairs for several organisms and is shown in Fig 6A. Comparing the gar (which did not experience the teleost specific genome duplication [26]) and the zebrafish, it can be seen that there is a slight increase in the number of Ohnologs in zebrafish (987 vs. 1352) (Fig 6). A slight increase in the average Ps (p = 0.03) value in the zebrafish is quite surprising since the zebrafish are thought to have experienced an additional genome duplication compared to the gar (Fig 6A). One of the most interesting results of this analysis, was that the average Ps value of human homeologous genes was quite low compared to all but the most recent genome duplication in Atlantic salmon (Fig 6A).

When plotted against the Atlantic salmon 4R genome duplication (Fig 6A), similar Ps values often cluster together. For example, chromosome three shares a large homeologous region with chromosome six and the Ps values in this region are typically between 0 and 0.1. The 3R genome duplication (which includes the 2R and 1R genome duplication in this analysis), does not show this clustering (S2 Fig). The Ps values of the 3R genome duplication, that overlap with the areas of high similarity in the 4R genome duplication, were not significantly (Welch's t-test, p = 0.99) different from the Ps values of the rest of the 3R genome duplication. This would support the hypothesis that any extra retention of ancient gene duplications is by chance because of the extra copies and not due to polysomic inheritance.

**Fig 7:**
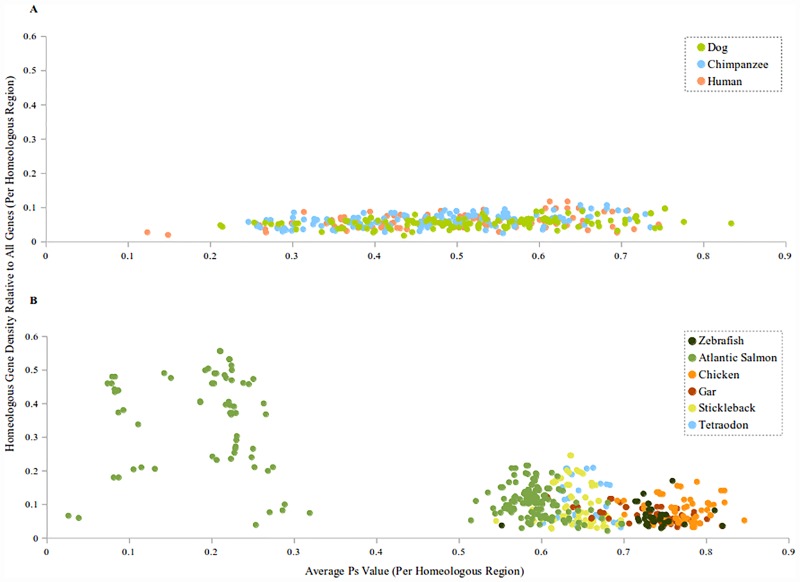
Scatter plot of homeologous regions from various vertebrate genomes. (A) Scatter plot of mammalian homeologous regions with the x-axis representing Ps values for the homeologous regions and the y-axis the corrected homeologous gene density for those regions. (B) Other vertebrate homeologous regions.

Fig 7 is a scatter plot of the Ps values versus the average homeologous gene density for all homeologous regions. The mammals have been separated from the rest of the organisms in this figure for clarity. The Ps values and the homeologous gene density all tightly cluster for the chicken, gar, stickleback, tetraodon, the Atlantic salmon 3R/Older genome, and zebrafish. For the human, dog, and chimpanzee genomes, the homeologous gene density is low, while the different homeologous regions have a large spread of Ps values (Fig 6). The Atlantic salmon has three distinct peaks at different Ps values, with the most dense regions having lower Ps values. The peak around Ps = 0.1, is composed of regions of the 4R genome that likely have multivalent pairing during meiosis, while the regions around the 0.23 peak contain the remaining regions of the 4R genome with likely fewer multivalent pairings during meiosis. The remaining peak contains all other regions associated with earlier genome duplications.

One piece of evidence supports the hypothesis that polysomic inheritance increases the odds of ancient gene duplication retention seen in Atlantic salmon. In Fig 2, it can be seen that there are more genes that have a salmonid specific genome gene copy (4R) as well as two additional gene copies from older genome duplications (3R/Older), than genes that have a salmonid specific genome gene copy and only a single additional gene copy from older genome duplications. If chance was the only mechanism maintaining the number of ancient gene copies, it is expected that there would be fewer genes that have a salmonid specific genome duplication gene copy as well as two additional gene copies from older genome duplications. In contrast, genes that have lost the 4R gene copy, there are much fewer genes that have retained two 3R/Older gene copies compared to those that have only retained one copy.

## Discussion

The two genome duplications, common to all vertebrates (1R and 2R), were clearly seen in the analysis of the human, gar, and chicken genomes in terms of expected number of homeologous chromosomes in the circos plots, but not in the difference between Ps values. The exact nature of these early duplications remains unclear because some chromosomes appear to have more and others to have fewer homeologous chromosomes than would be expected (i.e. if two duplications occurred, four homeologous chromosomes are expected, but often only three were observed). Plotting homeologous genes against a common chromosome (i.e. double conserved synteny) is better suited at distinguishing these relationships ([14], Fig 3)

Howe *et al*. (2013) produced a similar circos plot for the Zebrafish genome duplications, and in both their circos plot and in the current plot, multiple homeologous chromosomes can be seen for past genome duplications. It remains unclear, however, which of these homeologous chromosomes belong to the teleost specific genome duplication and which belong to earlier duplications.

Looking at quantitative data from these homeologous chromosomes does not improve the clarity of their origin. For example, the average Ps value between homeologous genes was slight, but significantly greater in the zebrafish genome than in the gar genome, possibly suggesting that the homeologous gene pairs in zebrafish had more time to accumulate silent mutations than the gene pairs in gar. The greater Ps value in the zebrafish could be mistaken as evidence that the genome duplication in zebrafish is older (because it appears to have had more time to accumulate silent mutations) than the genome duplication in gar.

A more likely interpretation (when considering double conserved synteny) of these results is that the Ps value was insufficient at discriminating between the teleost specific genome duplication and older genome duplications. The difference in Ps value may be considered irrelevant because mutational saturation has been reached and fluctuations in Ps values are not necessarily linked with time anymore. Another possibility is that the teleost specific genome duplication was allopolyploidy and homeologous genes were already diverged in the ancestor of zebrafish at the time of the teleost genome duplication.

Homeologous gene density values were similar to the Ps values, in that they were uninformative when distinguishing between the teleost specific and older genome duplications. Both the chicken and human genome duplications showed higher average homeologous gene densities than the zebrafish genome duplication, and the gar genome showed the same level of density as the zebrafish. This result is unlikely if the zebrafish genome duplication occurred after the vertebrate genome duplications.

The homeologous gene density information and the Ps values were useful for distinguishing the most recent genome duplication in the Atlantic salmon from more ancestral. The differences in Ps and density are quite consistent with the age estimates previously reported [2,10]. The teleost genome duplication (300 MYA) is ~3.75x the age of the salmonid specific genome duplication (80 MYA). The difference between the average Ps value for the teleost genome duplication (0.59) and the salmonid genome duplication (0.19) is also ~3x. The homeologous gene density follows the same trend of ~3x with the teleost genome duplication having a value of 0.10 and the salmonid genome duplication having a value of 0.32.

The Ps values and the homeologous gene density information was also useful for distinguishing the older genome duplications in the Atlantic salmon from the same duplications in zebrafish and other fish species. The older genome duplications in the Atlantic salmon appeared to have occurred earlier than the same duplications in the zebrafish. The Ps value was ~1.25x greater in the zebrafish genome duplications than in the same Atlantic salmon genome duplications and the homeologous gene density was ~2x greater in the Atlantic salmon genome duplications.

Several explanations are feasible for why the discrepancy between the zebrafish and the same Atlantic salmon genome duplications exists, but multivalent pairing of homeologous chromosomes during meiosis in Atlantic salmon offers a simple and well supported [8,27] basis for this difference. One expectation from this hypothesis is that genomic regions with greater levels of multivalent pairing would also have lower average Ps values between homeologous gene pairs when compared to the rest of the genome. Several regions of the Atlantic salmon genome appear to have consistently low Ps values between homeologous regions suggesting that they have higher levels of multivalent pairing than the rest of the genome. These areas of low divergence are expected based on predictions of multivalence pairing, and an approximate count of eight regions has been found by other researchers [8], which is consistent with the current count. Lien *et al*. (2011) found roughly the same chromosomes containing large numbers of multisite variants when compared to the rest of the genome [28], and again when sequencing the genome [4].

Another expectation from this hypothesis is that homeologous gene pairs from older genome duplications, in these high multivalent pairing regions, will have lower Ps values because they are being maintained by chromosome homogenization—similar to that seen for gene pairs from the most recent genome duplication. However, when tested, no difference was found for the Ps values between older homeologous gene pairs that overlapped with these regions of high multivalent pairing and those found throughout the rest of the genome.

This observation may be a bit misleading because the difference between the Ps values from ~80 MYA (the time when the salmonid genome was duplicated) and those found in the modern Atlantic salmon 3R genome duplication could be slight. Over 200 million years passed after the teleost genome duplication before the salmonid genome duplication occurred. Most of the variation seen in the synonymous substitution sites likely occurred during this time period, making any subsequent changes difficult to detect. Alternatively, if the teleost specific genome duplication was allopolyploidy, divergence may have already occurred between the two genomes at the time of duplication.

Taken together, four pieces of evidence: the Ps value peaks at 0.1 and 0.23, the lower average Ps value of the remaining Atlantic salmon homeologous regions, the greater retention of 3R gene copies that have a 4R gene copy as well (Fig 2), and previous observations of multivalent pairing of homeologous chromosomes [8], point to multivalent pairing being the likely reason that older genome duplications are better preserved in the Atlantic salmon than in other organisms. The only exception in these comparisons comes from the mammalian genomes. The average Ps values were lower in the human, chimpanzee, and dog genomes than that of the teleost specific genome duplication in Atlantic salmon.

The pattern of Ps values for the mammalian homeologous regions was quite different from the rest of the vertebrates compared. The gene density of the homeologous regions does not suggest that any of the lower Ps values represent genome duplications and based on the work of [14], the human genome has not experienced additional genome duplications besides the two common to vertebrates. Perhaps some of these regions are segmental duplications, but based on similar gene density among all the regions, this seems unlikely since it would mean that gene loss was more common than synonymous substitutions. This would suggest that in mammalian genomes duplicated gene pairs were maintained more so than in other vertebrate genomes.

Interestingly, the human X chromosome appears to have many homeologous regions throughout the genome. At least two examples are known where retrotransposed genes moved from the X chromosome to autosomes [29,30], possibly as a mechanism to evade X inactivation. This phenomenon may have enriched homeologous gene pairs by retaining them from genome duplications or from segmental duplications such as those caused by retrotransposition when they were derived from the X chromosome in the examples mentioned above.

By comparing genome duplications in various vertebrate genomes, we were able to identify large differences in the maintenance of gene duplicates between mammals and other vertebrates. Understanding this difference, may offer insight into the evolution of both groups. We were also able to better understand the salmonid specific genome duplication. It is difficult to overstate the importance of this genome duplication in the evolution and trajectory of salmonids. This duplication has increased the total gene count and with redundant gene copies has allowed genes to become specialized (subfunctionalization) and to have novel functions (neofunctionalization). In addition, it now appears that the 4R genome duplication has maintained gene copies from older genome duplications; perhaps allowing them more time to find a niche in the genomic landscape.

## Supporting information

S1 Fig Other vertebrate genome circos plot. Homeologous gene-pairs are connected by a line between chromosomes for the genomes. In the human genome, a large portion of the gene-pairs are between ribosomal proteins, however, when these are removed only the number of lines changes and not the relative patterns.
(TIF)

S2 Fig Proportion of silent substitutions (Ps) between homeologous gene pairs in ancient Atlantic salmon genome duplications. Circos plot of the homeologous regions from teleost specific (3R)/Older genome duplications with associated Ps values on the outer perimeter.
(TIF)

S1 Appendix Perl scripts used in analyses. These are the compressed scripts used to analyze the genome of several vertebrate species.
(GZ) Click here for additional data file.
