# Supplementary figures and images for: Autopolyploidy genome duplication preserves other ancient genome duplications in Atlantic salmon (Salmo salar)
Source: PLoS One. 2017 Feb 27;12(2):e0173053. doi: 10.1371/journal.pone.0173053 (PMC5328387; doi:10.1371/journal.pone.0173053)

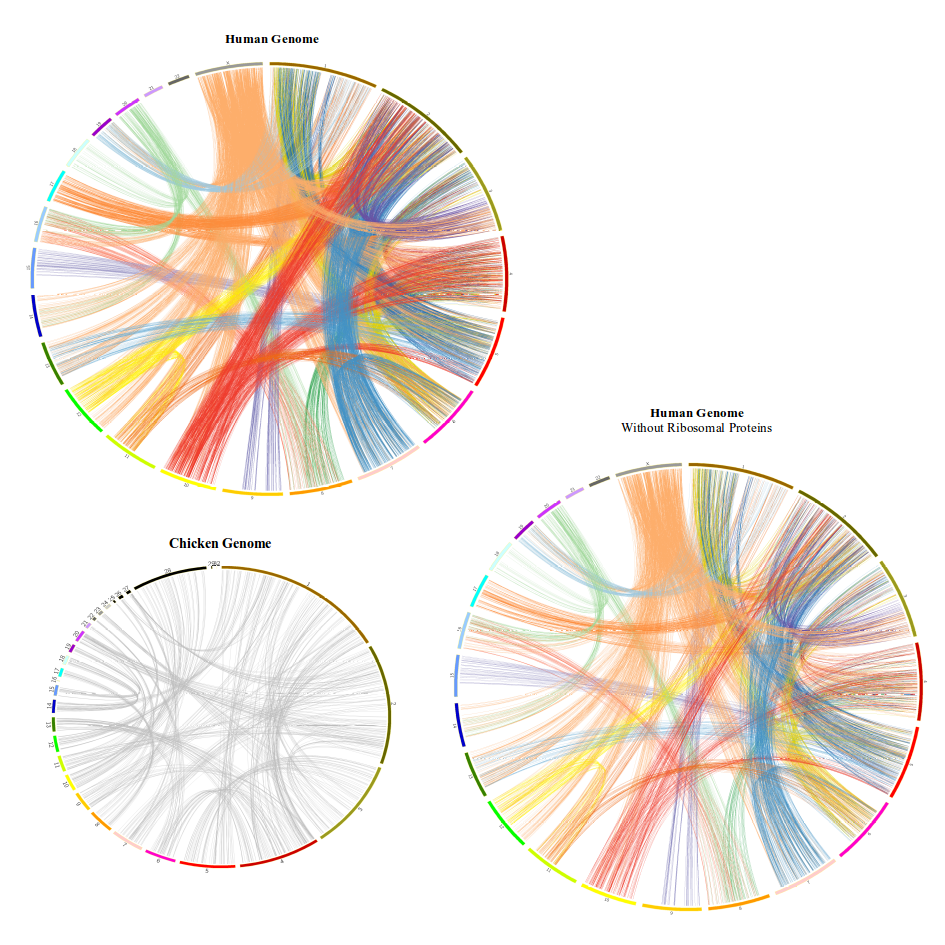

Supplement: S1 Fig — Homeologous gene-pairs are connected by a line between chromosomes for the genomes. In the human genome, a large portion of the gene-pairs are between ribosomal proteins, however, when these are removed only the number of lines changes and not the relative patterns. (TIF) [file pone.0173053.s001.tif]

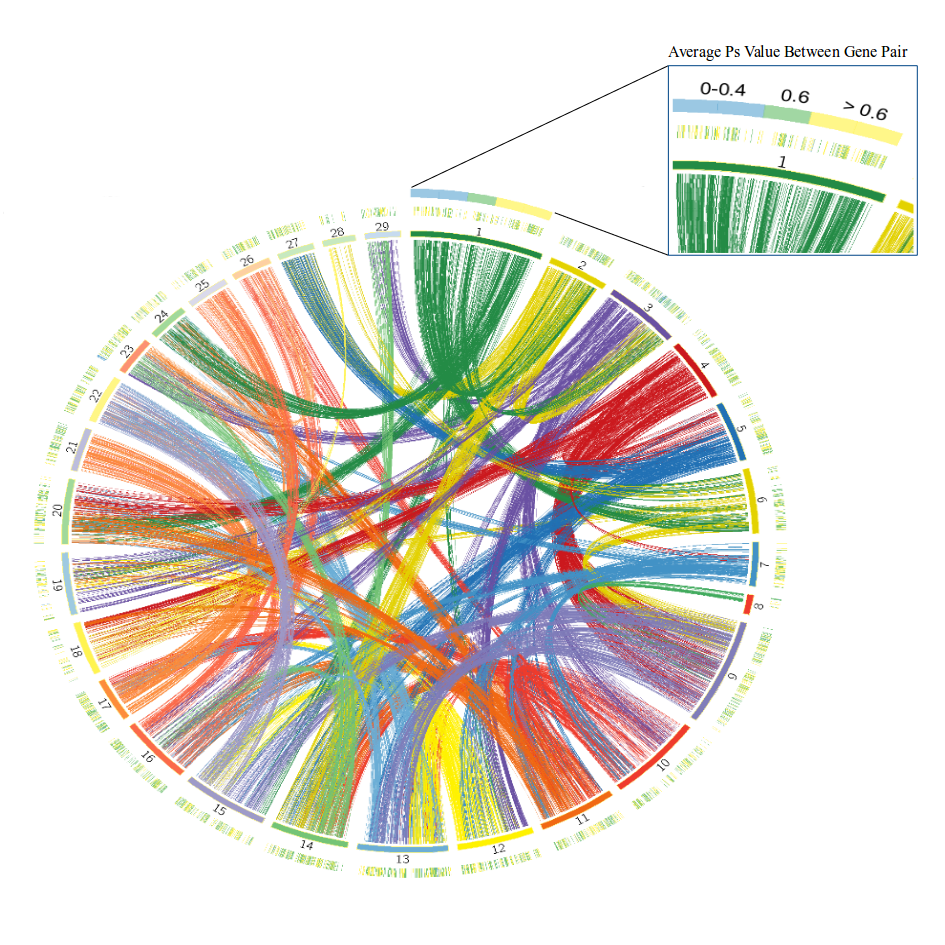

Supplement: S2 Fig — Circos plot of the homeologous regions from teleost specific (3R)/Older genome duplications with associated Ps values on the outer perimeter. (TIF) [file pone.0173053.s002.tif]
